# Habib*TM* 4X-assisted resection versus clamp-crush resection for hepatocellular carcinoma: a propensity-matching study

**DOI:** 10.18632/oncotarget.13906

**Published:** 2016-11-21

**Authors:** Jiliang Qiu, Weiqun Lu, Nanrong Yu, Guohua Yang, Yi Li, Zhiliang Huang, Jianchang Li, Kefei Li, Houwei Xu, Shicai Chen, Xiang Zeng, Haiying Liu

**Affiliations:** ^1^ Department of Abdominal Oncosurgery, Cancer Center of Guangzhou Medical University, Guangzhou, China; ^2^ Department of Surgery, the Fifth Affiliated Hospital of Sun Yat-Sen University, Zhuhai, China

**Keywords:** hepatocellular carcinoma, hepatic resection, Habib^TM^ 4X, clamp-crush, complication

## Abstract

Long term outcome of ablation-assisted hepatic resection is unclear for hepatocellular carcinoma (HCC) patients. This study was scheduled to compare the outcome of Habib 4X ablation assisted resection (Habib group) with clamp-crush resection (CC group) for HCC. In this study, we retrospectively enrolled 81 patients from the Habib group and 103 patients from the CC group. Oncologic outcomes were analyzed using a propensity score matching (PSM) method. Compared with the CC group, the Habib group had higher levels of γ-glutamyltransferase (*P*=0.044) and albumin (*P*=0.001), larger tumor sizes (*P*=0.007), shorter operation times (*P*=0.001), less blood loss (*P*=0.005), and less blood transfusions (*P*=0.038). There were no significant differences in complications (*P*=0.310), recurrence-free survival rates (RFS, *P*=0.112), or overall survival rates (OS, *P*=0.203) between the two groups. For the 67 patient pairs selected from the PSM analysis, the Habib group had better RFS and OS (*P*=0.033 and *P*=0.014, respectively). A Cox proportional hazards analysis revealed that Habib-assisted resection was an independent factor for RFS and OS (*P*=0.008 and *P*=0.016, respectively). Furthermore, for the 42 patients with central and large tumors, the Habib group had better RFS and OS than the CC group (*P*=0.035 and *P*=0.038, respectively). However, the differences of RFS and OS (*P*=0.117 and *P*=0.126, respectively) were not significant among 92 patients with peripheral or small tumors. Hence, Habib^TM^ 4X-assisted resection is safe and provides better survival for HCC patients, particularly those with central and large tumors.

## INTRODUCTION

Hepatocellular carcinoma (HCC) is the fifth most common cancer in the world. [1] It is well recognized that hepatic resection is the primary radical treatment for HCC. [2]

Recently, many new instruments, such as the TissueLink and Cavitron Ultrasonic Surgical Aspirator, have been developed to improve hepatic resections. [3] However, these techniques often require intraoperative maneuvers, including hepatic pedicle clamping, hypotensive anesthetics, which increased ischemic injury and risk of air embolism. [4]

The Habib^TM^ 4X, a newer bipolar radiofrequency ablation introduced in 2007, creates a plane of coagulative necrosis along the intended line of parenchymal transection. [5, 6] The Habib^TM^ 4X is a promising device for decreasing blood loss [7], without performing a Pringle maneuver. [8] To date, the safety and outcomes of patients who have undergone hepatic resection with the Habib^TM^ 4X have been controversial. [9, 10]

To further address this issue, we conducted this retrospective study to compare the Habib^TM^ 4X with clamp-crush in terms of complications, recurrence-free survival (RFS), and overall survival (OS) of patients. Furthermore, as a retrospective study, we used a propensity-scoring matched (PSM) model to balance the effects of variables before resection.

## RESULTS

### Patient characteristics

A total of 183 HCC patients who underwent hepatic resections were enrolled. Eighty-one (44.3%) patients were underwent resections with the Habib^TM^ 4X (Habib group), and 102 (55.7%) patients underwent clamp-crush resection (CC group). Overall, there were 159 (86.9%) male and 23 (13.1%) female patients. The median age was 51.0 years (range: 22-79 years). During a median follow-up of 41.5 months (range: 4.0-79.5 months), 91 patients (49.7%) experienced recurrence, and 62 patients (33.9%) died. The 2- and 5-year OS rates were 76.9% and 52.3%, and the 2- and 5-year RFS rates were 55.4% and 43.7%, respectively.

### Outcomes before matching

Table 1 summarizes preoperative and operative patient characteristics. Among the Habib group, 2 patients suffered bile leakage, 1 patient suffered hemorrhage, and 1 patient suffered liver failure. These were all major complications (Clavien-Dindo grade III-IV). Among the CC group, 3 patients developed bile leakage, 3 patients suffered hemorrhage, 2 patients suffered liver failure, and 1 patient suffered a severe lung infection, which were assigned as Clavien-Dindo grade III-IV (major complications). Neither group had dead within 30 days after resection. No significant differences in postoperative complications were observed between the two groups (*P* = 0.310).

**Table 1:** Clinicopathological factors of patients before and after propensity matched

	Before propensity matching			After propensity matching		
	Habib 4X group (n=81)	CC group (n=102)	*P* value	Habib 4X group (n=67)	CC group (n=67)	*P* value
Ages (years)	54.0±12.7	50±11.7	0.300	53.0±12.5	51.0±12.1	0.752
Gender (male: female)	69:12	90:12	0.544	58:9	58:9	1.000
HBsAg (positive : negative)	65:15	89:13	0.265	56:11	59:8	0.458
AFP (≤25: >25ng/ml)	34:47	34:68	0.229	27:40	22:45	0.370
White blood cell (X10^9/L)	6.1±2.2	6.5±2.1	0.732	5.9±2.2	6.2±2.2	0.699
Platelet count (X10^9/L)	167±91.1	151.5±87.7	0.453	166.0±71.7	145.0±99.5	0.930
ALT (U/L)	42.0±54.8	41.0±39.2	0.099	41.0±68.3	41.0±31.5	0.269
AST (U/L)	45.0±31.3	43.5±20.6	0.259	42.0±31.4	45.0±20.4	0.404
GGT (U/L)	54.0±22.9	50.0±17.1	0.044	51.0±51.7	53.0±27.4	0.355
Albumin (g/L)	38.5±7.5	41.0±4.2	0.001	38.8±7.7	40.6±4.5	0.007
Total bilirubin (umol/L)	18.6±14.7	16.0±6.9	0.063	16.5±14.8	16.0±6.8	0.229
Prothrombin time (s)	14.0±1.8	13.6±1.5	0.885	14.0±1.8	13.5±1.3	0.805
Ascites (absence : presence)	74:7	89:13	0.377	65:2	66:1	1.000
Tumor number (solitary : multiple)	60:21	71:31	0.506	49:18	50:17	0.844
Tumor size (cm)	6.5±2.7	5.5±4.0	0.007	5.5±2.4	5.0±3.1	0.463
Location (central : peripheral) ^a^	45:36	52:51	0.494	39:28	37:30	0.290
PVTT (absence : presence)	72:9	84:18	0.216	63:4	63:4	1.000
Pathological stage (I-II:III-IV)	39:42	51:51	0.803	32:35	39:28	0.226
Blood loss (ml)	150±572	250±674	0.005	175±533	250±467	0.037
Blood transfusion (no : yes)	71:10	77:25	0.038	60:7	49:18	0.015
Pringle maneuver (no : yes)	72:9	59:53	<0.001	63:4	38:29	<0.001
Surgical margin (<2 : ≥2 cm)	24:58	47:65	0.143	20:47	32:35	0.033
Operative time (minutes)	160±61	196±54	0.001	167±51	190±30	0.001
Complication grade (0-II: III-IV)	77:4	93:9	0.310	65:2	62:5	0.437
Hospital stays after surgery (days)	10.0±2.8	11.0±3.6	0.106	10.0±2.8	10.0±2.3	0.327

^a^ Central location is defined as tumor with a distance of ≥2cm from the liver capsule. The rest lesions are defined as peripheral.

Abrreviations: CC, clamp-crush, HBsAg, hepatitis B virus surface antigen; ALT, alanine aminotransferase; AST, aspartate aminotransferase; GGT, γ-glutamyl transferase; AFP, alpha-fetoprotein; PVTT, portal vein tumor thrombi.

**Figure 1:**
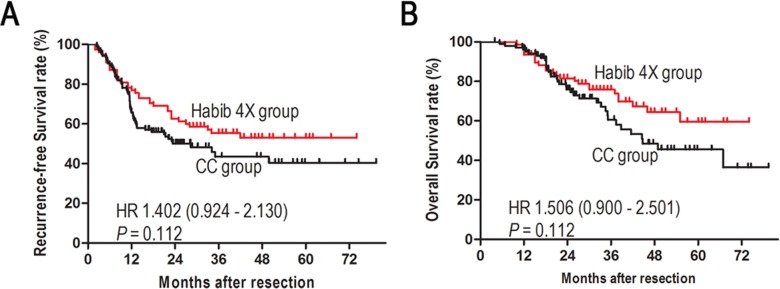
Kaplan-Meier analyses of recurrence-free survival **A**. and overall survival **B**. of patients with HCC before matching.

Compared with the CC group, the Habib group had higher levels of γ-glutamyltransferase (GGT; *P* = 0.044) and albumin (*P* = 0.001), larger tumors (*P* = 0.007), shorter operation times (*P* = 0.001), less intraoperative blood loss (*P* = 0.005), fewer intraoperative blood transfusions (*P* = 0.038), and fewer Pringle maneuvers (*P* = 0.001). The 2- and 5-year RFS rates were 61.2% and 52.8%, respectively, for the Habib group, and 50.1% and 40.2%, respectively, for the CC group (*P* = 0.112, Figure 1A). The 2- and 5-year OS rates were 80.5% and 56.2%, respectively, for the Habib group, and 73.2% and 45.5%, respectively, for the CC group (*P* = 0.112, Figure 1B).

### Outcomes after matching

**Figure 2:**
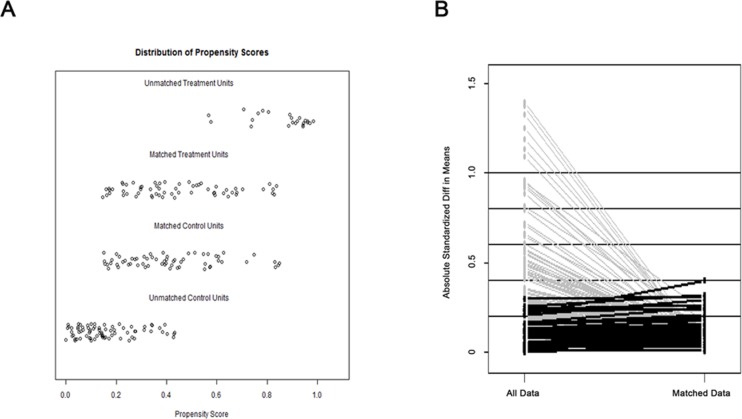
Parallel line plots of the standardized differences in means before and after PSM in patients with HCC **A**. Dot plots of the propensity scores of patients in the Habib and CC groups, showing individuals in the dataset and whether they were matched or discarded.

As mentioned before, the two groups have different baseline levels of several important preoperative clinical factors, including GGT and albumin levels as well as tumor size. Thus, we used the PSM method to balance bias. Using PSM, 67 paired patients were selected. The variable balance in the matched cohort was markedly improved, and no significant difference was observed between the two groups with respect to preoperative demographics (Table 1 and Figure 2). For the Habib group, 1 patient suffered bile leakage, and 1 patient suffered hemorrhage. For the CC group, 2 patients suffered bile leakage, 2 patients suffered hemorrhage, and 1 patient suffered liver failure. No significant difference in complications was observed between the two groups (*P* = 0.437).

Compared with the CC group, the Habib group had shorter operation times (*P* = 0.001), less blood loss (*P* = 0.037), blood transfusions (*P* = 0.015), and Pringle maneuvers (*P* = 0.001). During a median follow-up of 42.5 months (range: 4.0-74.0 months), 60 patients (44.8%) developed recurrence, and 41 patients (30.6%) died.

**Figure 3:**
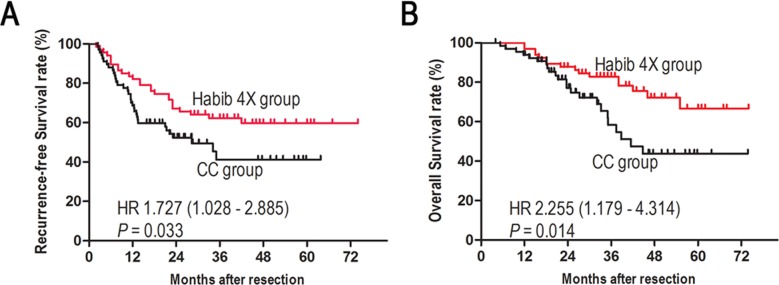
Kaplan-Meier analyses of recurrence-free survival **A**. and overall survival **B**. of patients with HCC after matching.

The associations of clinicopathological factors with RFS and OS are presented in Table 2. The 2- and 5-year RFS rates were 73.2% and 60.6%, respectively, in the Habib group, and 51.2% and 40.2%, respectively, in the CC group (*P* = 0.033, Figure 3A). The 2- and 5-year OS rates were 86.5% and 62.9%, respectively, in the Habib group, and 80.5% and 40.2%, respectively, in the CC group (*P* = 0.014, Figure 3B).

**Table 2:** Clinicopathological factor associated with the survival in matching patients

Variables	Recurrence-free survival rate (%)			Overall survival rate (%)		
	2y	5y	P value	2y	5y	P value
Gender						
Male: Female	49.7 : 60.2	49.7 : 52.1	0.605	78.4 : 79.6	55.0 : 55.6	0.776
Age (years) ^a^						
≤ 54 : >54	60.6 : 53.5	53.5 : 48.4	0.642	81.2 : 82.0	59.2 : 45.1	0.525
HBsAg						
Negative : Positive	61.7: 61.7	58.4 : 50.1	0.563	82.3 : 81.3	61.5 : 54.5	0.635
AFP level (ng/ml)						
≤ 25: > 25	59.1 : 57.7	53.3 : 49.9	0.953	78.4 : 80.6	50.8 : 59.6	0.895
ALT (U/L)						
≤40 : >40	59.2 : 58.5	52.6 : 50.6	0.526	85.0 : 78.3	56.1 : 55.6	0.446
AST (U/L)						
≤40 : >40	60.6 : 57.5	56.7 : 48.8	0.373	84.6 : 79.2	57.8 : 54.8	0.367
GGT (U/L)						
≤50 : >50	71.5 : 55.9	65.0 : 48.2	0157	91.6 : 79.0	78.6 : 49.4	0.039
Albumin						
≤ 35: > 35	58.3 : 58.8	43.2 : 52.9	0.124	70.0 : 80.5	36.9: 59.5	0.080
Total bilirubin (umol/L)						
≤17.1 : >17.1	58.3 : 65.8	53.2: 0	0.135	81.0 : 88.0	59.8 : 0	0.361
Ascites						
Absence : Presence	56.9 : 47.2	12.5 : 0	<0.001	82.6 : 33.3	55.9 : 33.3	0.095
Prothrombin time (s)						
<14 : >14	57.4 : 62.3	52.1 : 51.0	0.954	79.7 : 85.9	53.3 : 62.2	0.541
Tumor number						
Solitary : Multiple	60.4 : 57.8	51.2 : 46.2	0.356	82.1 : 79.1	56.3 : 54.5	0.723
Tumor size (cm)						
≤5 : >5	68.4 : 52.2	68.4 : 40.4	0.012	86.5 : 73.0	63.5 : 49.3	0.088
Location						
Central : Peripheral	56.6 : 61.6	49.0 : 54.8	0.515	86.9 : 77.3	55.3 : 53.7	0.303
PVTT						
Absence : Presence	61.7 : 12.5	55.2 : 0	<0.001	83.6 : 46.8	60.9 : 0	<0.001
Blood loss (ml) ^a^						
≤200 : >200	59.1 : 57.7	50.9 : 54.4	0.890	83.1 : 71.5	55.3 : 57.8	0.446
Blood transfusion						
No : yes	61.5 : 22.0	53.8 : 0	0.018	83.6 : 50.8	57.4 : 0	0.014
Surgical margin (cm)						
<2 : ≥2	51.8 : 61.8	37.5 : 55.6	0.129	71.4 : 85.3	53.5 : 57.4	0.143
Resection approach						
CC group : Habib4x group	52.3 : 65.6	41.2 : 59.5	0.033	72.8 : 86.5	44.2 : 61.8	0.030
Pathological stage						
I-II : III-IV	59.9 : 56.9	53.5 : 49.0	0.582	80.4 : 82.8	48.8 : 66.4	0.282

a Patients were divided according to the median value.

Abrreviations: CC, clamp-crush, HBsAg, hepatitis B virus surface antigen; ALT, alanine aminotransferase; AST, aspartate aminotransferase; GGT, γ-glutamyl transferase; AFP, alpha-fetoprotein; PVTT, portal vein tumor thrombi.

**Table 3:** Cox’s regression analysis in matching patients

Variables	Recurrence-free survival		Overall survival	
	HR (95.0% CI)	*P* value	HR (95.0% CI)	*P* value
Albumin (≤35:>35 g/L)	-	-	2.857 (1.372-5.952)	0.005
Ascites (Absence: Presence)	-	-	0.206 (0.047-0.900)	0.036
Resection approach (Habib : CC)	0.488 (0.287-0.829)	0.008	0.449 (0.234-0.860)	0.016
Tumor size (≤5 : > 5 cm)	0.472 (0.268-0.831)	0.009	-	-
PVTT (absence : presence)	0.180 (0.083-0.392)	<0.001	0.186 (0.079-0.439)	<0.001

Abbreviation: HR, hazard rate; CI, confidence interval; CC, clamp-crush; PVTT, portal vein tumor thrombosis.

PVTT (hazard ratio [HR], 0.180; 95% confidence interval [CI] 0.083-0.392; *P* < 0.001), tumor size (HR, 0.472; 95% CI: 0.268-0.831; *P* = 0.009), and resection method (HR, 0.488; 95% CI: 0.287-0.829; *P* = 0.008) were independent predictive factors of RFS, after adjusting for propensity score. PVTT (HR, 0.186; 95% CI: 0.079-0.439; *P* < 0.001), albumin (HR, 2.857; 95% CI: 1.372-5.952; *P* = 0.005), resection method (HR, 0.449; 95% CI: 0.234-0.860; *P* = 0.016), and ascites (HR, 0.206; 95% CI: 0.047-0.900; *P* = 0.036) were independent prognostic factors for OS, after adjusting for propensity score.

**Figure 4:**
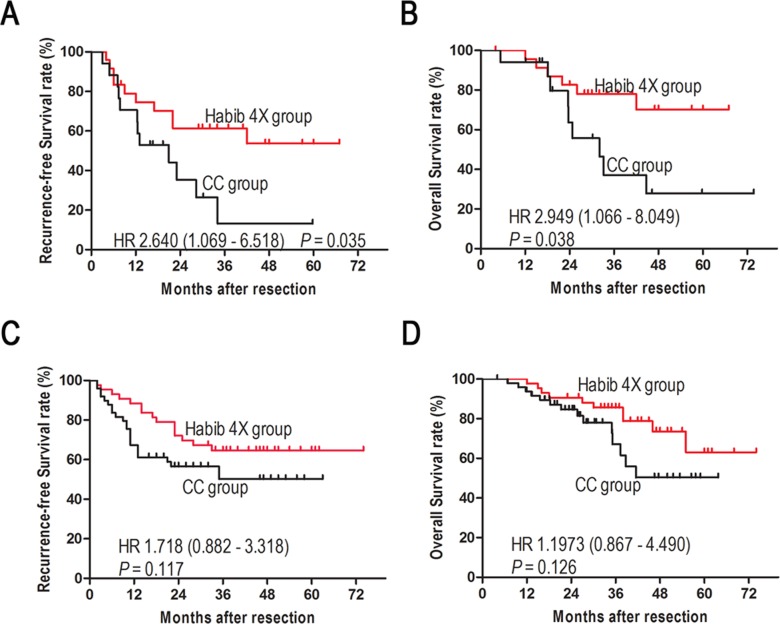
After matching, Kaplan-Meier analyses of recurrence-free survival **A**. and overall survival **B**. of 42 patients with central and large HCCs. Kaplan-Meier analyses of recurrence-free survival C. and overall survival D. of 92 patients with peripheral or small HCCs.

In addition, resections of central tumors [11] and large tumors [12] were always associated with more blood loss and poor survival. Thus, we further stratified patients according to tumor location and tumor size. Central tumors were defined as previous: tumor located central segments (Couinaud’s segments I, IV, V and VIII) and with a distance of ≥ 2 cm from the liver capsule. [11] For the 42 patients with central and large tumors (tumor size more than 5 cm), the Habib group had better RFS and OS (*P* = 0.035 and *P* = 0.038, respectively, Figures 4A and 4B). While, for the 92 patients with peripheral and small tumors (tumor size no more than 5 cm), two groups had similar RFS and OS (*P* = 0.117 and *P* = 0.126, respectively, Figure 4C and 4D).

## DISCUSSION

Although the safety and survival of patients who undergo ablation-assisted resections has been evaluated, [8, 13] there is still lack of results regarding the oncological outcome of Habib^TM^ 4X-assisted resections. As the best of our knowledge, this is the first study to investigate the long term oncological outcome due to the technique of resection. In the current study, patients in the Habib group had similar survival rates as those in the CC group before matching. After one-to-one PSM analysis, the Habib group was significantly associated with less blood loss, lower rates of blood transfusion, and shorter operation times, compared with the CC group. The Habib group had better RFS and OS compared to the CC group. In addition, patients with central or large tumors are better candidate for Habib^TM^ 4X-assisted resections, compared to the clamp-crush resection. This study supports the Habib^TM^ 4X providing safer resections and favorable survival for HCC patients.

During the past decade, there has been a significant increase in the number of liver resections. [14, 15] Still, resection comes with significant risks and the high frequency of complications. [16] In our study, the rates of severe complications before and after matching were 4.9% and 3.0%, respectively, for the Habib group, which were both relatively lower than patients in the CC group and previous studies. [17, 18] This indicates that Habib^TM^ 4X-assisted resection is a safe.

CC is recognized as a standard method of liver parenchymal transection over the past decades. However, CC often lead to excessive blood loss and more blood transfusions, which further influenced the safety and survival. [19] The Habib^TM^ 4X releases energy and produces a plane of coagulative necrosis along the intended line of parenchymal transection. [20] As this process reduce the risk of bleeding of hepatic parachymal, it thus ensured rapid hepatic parachymal division and shorted operation times. This may benefit cirrhotic patients. The rate of required blood transfusions was 12.3% (10/81), which was much lower than that for most previous studies. [21, 22]

Our data revealed that Habib^TM^ 4X-assisted resection provided better survival than the traditional clamp-crush method. One of the potential reasons is the coagulative necrosis zone formed by the Habib^TM^ 4X. This ablation zone was a tumor cell-free zone, which could kill potentially metastatic cells and decrease the risk of local recurrence. A clinical trial showed that narrow surgical margins increased intrahepatic recurrence [23]. Furthermore, a coagulative necrosis zone was made before the hepatic resection could reduce the risk of tumor cell intravasation and dissemination into the circulation. In addition, recently studies showed that after thermal ablation-mediated necrosis, tissue debris remains in the treated area, which may help active tumor-specific T cell response and increase the likelihood of tumor control. [24–26]

Studies from the team of Curro and Habib opened an eye for liver surgeon to recognize the Habib^TM^ 4X device, [27, 28] while there are still many problems need to be discussed, including long term oncological outcome. [29] An Italian study showed that ablation-assisted resection provides a clean surgical field, but it was associated with a higher rate of complications than clamp-crushing. [9] Recently, another study showed that ablation-assisted resection resulted in lower blood loss and fewer complications. [10] These two studies have controversial outcomes, and a meta-analysis was also unable to reach convincing outcomes. [30] While both of these studies had small sample sizes, lacked oncologic outcomes, and were based on monopolar ablation. [9, 10] We surmised that the HabibTM 4X, a bipolar ablation device, produce a better coagulative necrosis zone than with monopolar ablation. [31]

It is interesting to note that the Habib^TM^ 4X may provide favorable survival rates for central and large tumors. Resection for central and large tumors are always associated more blood loss and longer operation time. The advantage of Habib^TM^ 4X-assisted resection is more obviously for central and large tumors. While, the benefits of Habib^TM^ 4X over clamp-crush was unclear for peripheral or small tumors Thus, patients with central and large tumors were good candidate for Habib^TM^ 4X-assisted resection.

This study had limitations. Thermal damage from ablation may injure the neighboring vessels. Thus, the Habib^TM^4X requires skilled surgical techniques. Use of ultrasound guidance greatly decreases the risk of injury to large vessels. The Habib^TM^ 4X device should be operated by experienced surgeons. In our study, all resections were performed by Haiying Liu, who has performed at least 80 hepatic resection per year. Furthermore, with increased availability and practice of laparoscopy, laparoscopic HabibTM 4X procedures could greatly control blood loss. [20, 32] We have performed several hepatic resections with laparoscopic Habib^TM^ 4X. A large sample size study could provide more specific information on laproscopic Habib^TM^ 4X in the near future. In addition, a comparative study of Habib^TM^ 4X with other surgical tools, such as bipolar scissors and LigaSure diathermy, may broaden our understanding of Habib^TM^ 4X and increase hepatic resection device options. Finally, it should be noted that this is a retrospective and single-institution study. A prospective, multi-center study is needed to validate our findings.

## CONCLUSIONS

In conclusion, Habib^TM^ 4X-assisted hepatic resection provides safety and survival benefits for patients with HCC who undergo hepatic resections, particularly for those with central and large tumors.

## MATERIALS AND METHODS

### Study population

Between January 2009 and December 2013, 183 patients underwent hepatic resections at Guangzhou Medical University Cancer Center. This retrospective study was approved by the Institutional Review Board at Guangzhou Medical University Cancer Center. All the patients were diagnosed with HCC. None had undergone liver transplantation, ablation, trans-arterial chemoembolization (TACE), or radiotherapy before resection.

### Operative techniques

Patients underwent liver resections with general anesthesia. Intraoperative bimanual liver palpation and ultrasonography were then performed to confirm tumor and major vessel locations. If a resection plane was close to a secondary major vessel, resection was performed using the clamp-crush technique (CC group), which divides and seals the hepatic duct. If tumors were distal ( ≥ 1 cm) to the secondary major or major vessels, the Habib^TM^ 4X device was used to develop a coagulation plane and create a resection line with an argon diathermy 1-2 cm from the edge of the tumor (Habib group). For each application, 100 W were delivered, and the procedure took 5-15 s. According to the thickness and vascular distribution of the liver tissue, we repeated the above steps until a fully ablated zone of desiccation was created. The number of ablations required to obtain a zone of necrosis depended on the depth of the liver parenchyma to be resected. This zone of desiccation was related to the size of the cut resection margin surface.

Once ablation was completed, a scalpel was used to divide the parenchyma between the pair of needles. Following that, we ligated the large blood vessels and bile ducts with diameters larger than 7 mm, leaving only the coagulated liver parenchyma behind. These processes were repeated until the entire tumor was removed.

### Follow-up

In this study, the Clavien-Dindo classification was used to accurately and objectively grade the severity of postoperative complications. [33, 34] Patients underwent contrast-enhanced CT or magnetic resonance imaging (MRI) of the abdomen and chest x-ray every 3 months for the first two years after resection and every 5-6 months subsequently for follow-up. Serum alpha-fetoprotein (AFP) levels and liver function were tested simultaneously. Recurrence diagnoses were made based on imaging alone if the tumor displayed typical enhancement characteristics. Extrahepatic tumors or those with atypical imaging characteristics were biopsied to confirm HCC. The data in this study were censored on February 1, 2016. Whenever possible, salvage treatment was administered to patients with recurrence or metastases. Repeat resections and ablations were the primary treatment choices for patients with solitary lesions or up to 3 lesions in total. Other non-radical treatments included transarterial chemoembolization (TACE).

### Statistical analysis

Statistical analyses were performed using SPSS 20.0 (IBM, New York, NY, USA) and R version 2.12.1. OS was defined as the time from the date of resection to the date of death or the last follow-up. RFS was defined as the time from the date of resection to the time of recurrence, metastasis, or the last follow-up. RFS and OS rates were generated using the Kaplan-Meier method with log rank analyses. The Cox regression model was built using a stepwise variable selection.

PSM analysis was performed as described in our previous study [35]. Age, sex, tumor size, tumor number, tumor location, portal vein tumor thrombosis (PVTT), levels of alanine aminotransferase (ALT), aspartate aminotransferase (AST), γ-glutamyltransferase (GGT), albumin, total bilirubin, and ascites were the variables analyzed. Subsequently, 1:1 matching between the CC group and the Habib group was performed using the nearest neighbor matching. Once patients were matched, conditional logistic regression was used to compare survival. All *P* value calculations were 2-sided, and *P* < 0.05 was considered statistically significant.

## References

[1] Singal AG, El-Serag HB (2015). Hepatocellular Carcinoma From Epidemiology to Prevention: Translating Knowledge into Practice. Clin Gastroenterol Hepatol.

[2] Ulahannan SV, Duffy AG, McNeel TS, Kish JK, Dickie LA, Rahma OE, McGlynn KA, Greten TF, Altekruse SF (2014). Earlier presentation and application of curative treatments in hepatocellular carcinoma. Hepatology.

[3] Wong JS, Lee KF, Cheung YS, Chong CN, Wong J, Lai PB (2011). Modification of right hepatectomy results in improvement outcome: a retrospective comparative study. HPB (Oxford).

[4] Koo BN, Kil HK, Choi JS, Kim JY, Chun DH, Hong YW (2005). Hepatic resection by the Cavitron Ultrasonic Surgical Aspirator increases the incidence and severity of venous air embolism. Anesth Analg.

[5] Stavrou GA, Tzias Z, von Falck C, Habib N, Oldhafer KJ (2007). Hepatic resection using heat coagulative necrosis. First report of successful trisegmentectomy after hypertrophy induction. Langenbecks Arch Surg.

[6] Dagher I, O’Rourke N, Geller DA, Cherqui D, Belli G, Gamblin TC, Lainas P, Laurent A, Nguyen KT, Marvin MR, Thomas M, Ravindra K, Fielding G (2009). Laparoscopic major hepatectomy: an evolution in standard of care. Ann Surg.

[7] Curro G, Habib N, Jiao L, Baccarani U, Scisca C, Navarra G (2008). Radiofrequency-assisted liver resection in patients with hepatocellular carcinoma and cirrhosis: preliminary results. Transplant Proc.

[8] Pai M, Frampton AE, Mikhail S, Resende V, Kornasiewicz O, Spalding DR, Jiao LR, Habib NA (2012). Radiofrequency assisted liver resection: analysis of 604 consecutive cases. Eur J Surg Oncol.

[9] Lupo L, Gallerani A, Panzera P, Tandoi F, Di Palma G, Memeo V (2007). Randomized clinical trial of radiofrequency-assisted versus clamp-crushing liver resection. Br J Surg.

[10] Li M, Zhang W, Li Y, Li P, Li J, Gong J, Chen Y (2013). Radiofrequency-assisted versus clamp-crushing parenchyma transection in cirrhotic patients with hepatocellular carcinoma: a randomized clinical trial. Dig Dis Sci.

[11] Qiu J, Wu H, Bai Y, Xu Y, Zhou J, Yuan H, Chen S, He Z, Zeng Y (2013). Mesohepatectomy for centrally located liver tumours. Br J Surg.

[12] Cai ZQ, Si SB, Chen C, Zhao Y, Ma YY, Wang L, Geng ZM (2015). Analysis of prognostic factors for survival after hepatectomy for hepatocellular carcinoma based on a bayesian network. PLoS One.

[13] Pai M, Kyriakides C, Mikhail S, Habib N, Spalding D, Jiao L, Cherqui D (2011). Radiofrequency-assisted hepatic resection. Ann Surg Oncol.

[14] Ishizawa T, Mise Y, Aoki T, Hasegawa K, Beck Y, Sugawara Y, Kokudo N (2010). Surgical technique: new advances for expanding indications and increasing safety in liver resection for HCC: the Eastern perspective. J Hepatobiliary Pancreat Sci.

[15] Zeng QA, Qiu J, Hong J, Li Y, Li S, Zou R, Huang P, Li B, Zheng Y, Lao X, Yuan Y (2012). Hepatectomy for hepatocellular carcinoma patients with macronodular cirrhosis. Eur J Gastroenterol Hepatol.

[16] Harimoto N, Shirabe K, Ikegami T, Yoshizumi T, Maeda T, Kajiyama K, Yamanaka T, Maehara Y (2015). Postoperative complications are predictive of poor prognosis in hepatocellular carcinoma. J Surg Res.

[17] Yamashita Y, Ikeda T, Kurihara T, Yoshida Y, Takeishi K, Itoh S, Harimoto N, Kawanaka H, Shirabe K, Maehara Y (2014). Long-term favorable surgical results of laparoscopic hepatic resection for hepatocellular carcinoma in patients with cirrhosis: a single-center experience over a 10-year period. J Am Coll Surg.

[18] Yin Z, Fan X, Ye H, Yin D, Wang J (2013). Short- and long-term outcomes after laparoscopic and open hepatectomy for hepatocellular carcinoma: a global systematic review and meta-analysis. Ann Surg Oncol.

[19] Huang G, Lai EC, Lau WY, Zhou WP, Shen F, Pan ZY, Fu SY, Wu MC (2013). Posthepatectomy HBV reactivation in hepatitis B-related hepatocellular carcinoma influences postoperative survival in patients with preoperative low HBV-DNA levels. Ann Surg.

[20] Akyildiz HY, Morris-Stiff G, Aucejo F, Fung J, Berber E (2011). Techniques of radiofrequency-assisted precoagulation in laparoscopic liver resection. Surg Endosc.

[21] Yang T, Lu JH, Lau WY, Zhang TY, Zhang H, Shen YN, Alshebeeb K, Wu MC, Schwartz M, Shen F (2016). Perioperative blood transfusion does not influence recurrence-free and overall survivals after curative resection for hepatocellular carcinoma: A Propensity Score Matching Analysis. J Hepatol.

[22] Zhu P, Zhang B, Wang R, Mei B, Cheng Q, Chen L, Wei G, Xu DF, Yu J, Xiao H, Zhang BX, Chen XP (2015). Selective Inflow Occlusion Technique versus Intermittent Pringle Maneuver in Hepatectomy for Large Hepatocellular Carcinoma: A Retrospective Study. Medicine (Baltimore).

[23] Shi M, Guo RP, Lin XJ, Zhang YQ, Chen MS, Zhang CQ, Lau WY, Li JQ (2007). Partial hepatectomy with wide versus narrow resection margin for solitary hepatocellular carcinoma: a prospective randomized trial. Ann Surg.

[24] Shi Y, Evans JE, Rock KL (2003). Molecular identification of a danger signal that alerts the immune system to dying cells. Nature.

[25] Mizukoshi E, Yamashita T, Arai K, Sunagozaka H, Ueda T, Arihara F, Kagaya T, Yamashita T, Fushimi K, Kaneko S (2013). Enhancement of tumor-associated antigen-specific T cell responses by radiofrequency ablation of hepatocellular carcinoma. Hepatology.

[26] Cui J, Wang N, Zhao H, Jin H, Wang G, Niu C, Terunuma H, He H, Li W (2014). Combination of radiofrequency ablation and sequential cellular immunotherapy improves progression-free survival for patients with hepatocellular carcinoma. Int J Cancer.

[27] Curro G, Bartolotta M, Barbera A, Jiao L, Habib N, Navarra G (2009). Ultrasound-guided radiofrequency-assisted segmental liver resection: a new technique. Ann Surg.

[28] Curro G, Jiao L, Scisca C, Baccarani U, Mucciardi M, Habib N, Navarra G (2008). Radiofrequency-assisted liver resection in cirrhotic patients with hepatocellular carcinoma. J Surg Oncol.

[29] Curro G, Navarra G (2016). Ultrasound-guided radiofrequency-assisted segmental liver resection. Surgery.

[30] Xiao WK, Chen D, Hu AB, Peng BG, Guo YZ, Fu SJ, Liang LJ, Li SQ (2014). Radiofrequency-assisted versus clamp-crush liver resection: a systematic review and meta-analysis. J Surg Res.

[31] Cartier V, Boursier J, Lebigot J, Oberti F, Fouchard-Hubert I, Aube C (2016). Radiofrequency ablation of hepatocellular carcinoma: Mono or multipolar?. J Gastroenterol Hepatol.

[32] Gadiyaram S, Shetty N (2012). Laparoscopic resection of giant liver hemangioma using laparoscopic Habib probe for parenchymal transection. J Minim Access Surg.

[33] Clavien PA, Barkun J, de Oliveira ML, Vauthey JN, Dindo D, Schulick RD, de Santibanes E, Pekolj J, Slankamenac K, Bassi C, Graf R, Vonlanthen R, Padbury R (2009). The Clavien-Dindo classification of surgical complications: five-year experience. Ann Surg.

[34] Dindo D, Demartines N, Clavien PA (2004). Classification of surgical complications: a new proposal with evaluation in a cohort of 6336 patients and results of a survey. Ann Surg.

[35] Qiu J, Zheng Y, Shen J, Zeng QA, Zou R, Liao Y, He W, Li Q, Chen G, Li B, Yuan Y (2015). Resection versus ablation in hepatitis B virus-related hepatocellular carcinoma patients with portal hypertension: A propensity score matching study. Surgery.

